# Association between triglyceride glucose index and risk of cancer: A meta-analysis

**DOI:** 10.3389/fendo.2022.1098492

**Published:** 2023-01-12

**Authors:** Huan Wang, Feifei Yan, Yani Cui, Feinan Chen, Guixia Wang, Weiwei Cui

**Affiliations:** ^1^ Department of Endocrinology and Metabolism, First Hospital of Jilin University, Changchun, China; ^2^ Department of Nutrition and Food Hygiene, School of Public Health, Jilin University, Changchun, China

**Keywords:** triglyceride glucose index, cancer, observational research, random effects model, meta-analysis

## Abstract

**Background:**

Triglyceride glucose (TyG) index as a more convenient and reliable predictor of insulin resistance (IR) is thought to be associated with many diseases, but its relationship with cancer remains unclear.

**Methods:**

The meta-analysis was conducted to evaluate the effects of TyG index on cancer risk utilizing the available evidence. PubMed, EMBASE, Medline, Cochrane Library and Web of Science were searched from their inception up to July 2022. A random-effects model was used to calculate the effect estimates and 95% confidence intervals (CIs).

**Results:**

A total of 6 observational studies met our inclusion criteria, which including 992292 participants. The meta-analysis indicated that the higher TyG index increased cancer risk compared to the lower TyG index group (total effect size =1.14, 95% CI [1.08, 1.20], *P*<0.001).

**Conclusions:**

Our meta-analysis found that higher TyG index may increase the risk of cancer. More prospective cohort studies and basic research are warranted to verify the relationship.

## 1 Introduction

Cancer is the result of a serious disruption in the regulation of cell growth and proliferation, usually manifesting as a local abnormal tissue mass in the body (1, 2). Cancer can not only infiltrate and grow in the primary site and involve adjacent organs or tissues, but also spread to other parts of the body through a variety of ways (lymphatic metastasis, hematogenous metastasis and seeding metastasis), which will seriously increase its harmfulness (3, 4). At present, cancer incidence and mortality are increasing rapidly all over the world, posing a major challenge to society, healthcare systems, and patients and their families. The Global Burden of Cancer Report estimates that 19.3 million new cancer cases will be found and 10.0 million people will die from cancer in 2020, overtaking heart disease as the second common cause of death in the world (5, 6). According to the latest projections, an estimated 28.4 million new cancer cases will occur globally in 2040, a 47% increase from the corresponding 19.3 million cases in 2020 (5). Cancer will become the world’s leading cause of death and the greatest obstacle of extending life expectancy in the 21st century (7). Fortunately, recent studies have manifested that the elimination or reduction of known unhealthy lifestyles and environmental risk factors could prevent one-third to two-fifths of new cancer patients (8, 9). For example, people who often consume rich saturated fat and refined sugar in their daily life might increase body fat accumulation and impaired glucose and insulin regulation, which in turn altered physiological hormonal homeostasis and ultimately increased cancer risk (10, 11).

The formation of cancer is a complex process, the result of severe disturbances in the regulation of cell growth and proliferation. Recently, emerging evidence confirmed that Insulin resistance (IR), partly regulated by diet and lifestyle (12, 13), was strongly associated with the morbidity and mortality of various cancers, including colorectal cancer, breast cancer and lung cancer (14–18). This phenomenon might be due to the fact that IR directly increases cell proliferation and inhibits cell apoptosis, activates insulin-like growth factor (IGF-1) receptors or triggers oxidative stress and inflammatory processes, thereby promoting the occurrence and development of cancer (10, 19–22).

It is well known that the Homeostatic Model Assessment of Insulin Resistance and hyperinsulinemic-euglycemic clamp test can measure IR effectively (23, 24). Moreover, several studies have demonstrated that triglyceride glucose (TyG) index has been considered to be a more convenient and reliable predictor of IR compared to these two common measurement tools (23, 25). Furthermore, the TyG index has been reported to be closely related to the occurrence of cancer (26, 27). Nevertheless, recent studies have also shown that the cancer risk was not affected by the TyG index (28, 29). The role of TyG index in cancer risk remains controversial and its relationship has not been demonstrated in meta-analysis. Therefore, we performed a meta-analysis of all relevant observational studies focusing mainly on the association of cancer risk with TyG index.

## 2 Methods

### 2.1 Sources and methods of data retrieval

Our meta-analysis was conducted in accordance with the PRISMA guidelines and extensions (30) and the specific details are shown in **Table S1**. The electronic databases including PubMed, EMBASE, Web of Science, Medline and Cochrane Library were searched from the inception up to July 2022. The following terms (combined with the Boolean logical operator ‘OR.’ or ‘AND’) were used for the literature search: cancer, tumor, neoplasms [MeSH Terms], malignant neoplasm, carcinoma [MeSH Terms], triglyceride glucose index, TyG index, triglyceride glucose indices and Triglyceride/glucose index. Terms such as population, language and study design were not restricted in the literature search. The specific search strategies are listed in **Table S2**.

### 2.2 Inclusion and exclusion criteria

**Figure 1:**
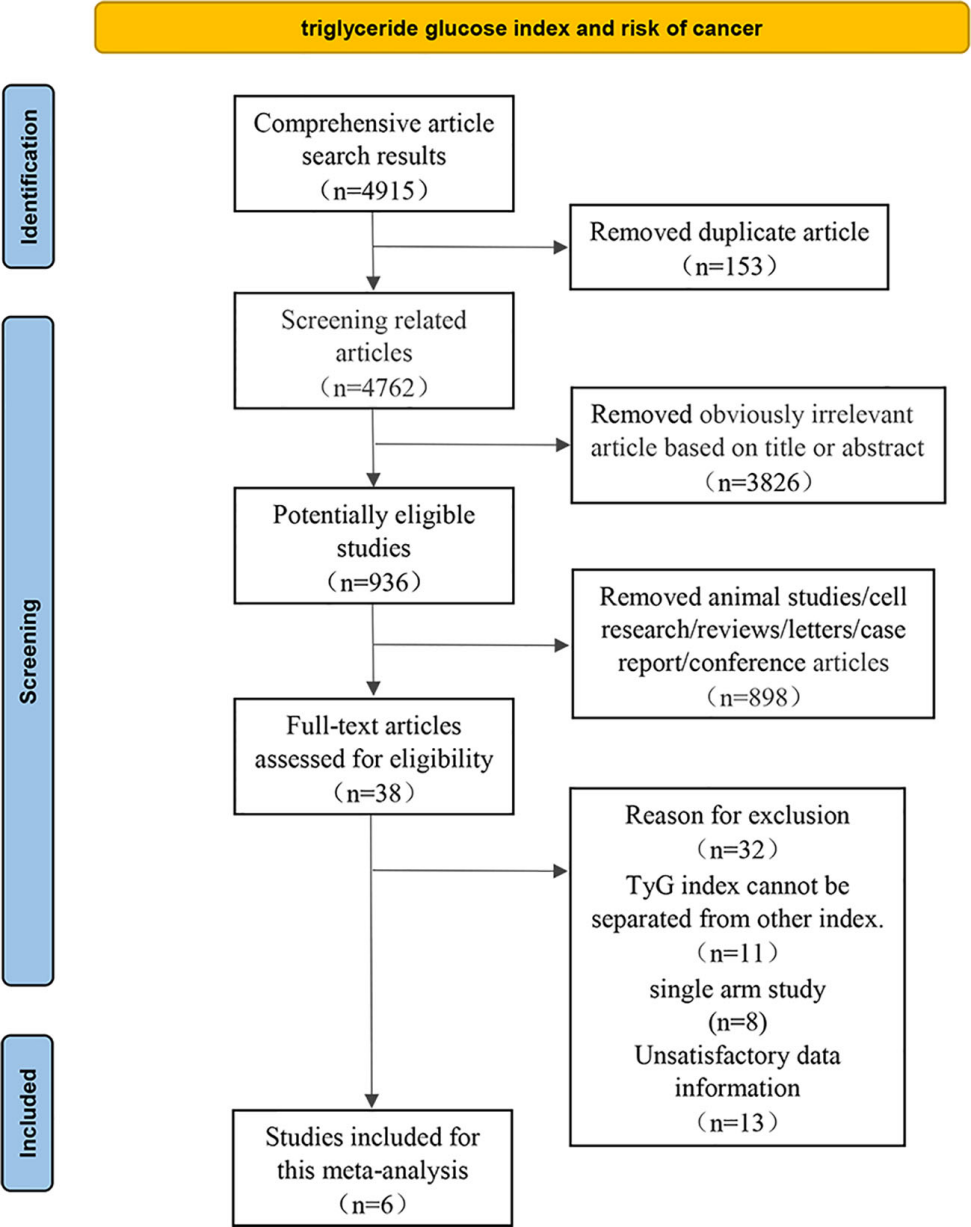
Flow diagram of the literature search and selection.

The eligible studies were based on the following criteria (1): the study design was observational study; (2) TyG index could be obtained *via* laboratory examination and cancer in a specific anatomical site was clearly defined as an outcome indicator; (3) Association between TyG index and cancer risk was presented by odds ratios (ORs) or hazard ratios (HRs) along with their 95% confidence intervals (CIs), or comprehensive data was provided to calculate them; Meanwhile, we excluded some studies such as *in vitro* studies, animal experiments, duplicate literature, reviews, letters, case report or conference papers. Two researchers independently reviewed all relevant studies, extracted potentially eligible data, fully discussed and resolved uncertainty and disagreements (**Figure 1**).

### 2.3 Data abstraction

We extracted the following crucial data among all included relevant studies: (1) first author, publication year, the nationality of subjects, study design, cancer site, sample size, mean age and gender of participant. (2) TyG index levels in different groups. (3) Adjusted total cancer risk estimates (OR or HR) and their corresponding 95% CIs.

### 2.4 Quality assessment

The quality of the observational literature was assessed independently by two investigators using the Newcastle-Ottawa scale (NOS) (31). This scale consists of three parts with a total of 9 points. Quality scores of greater than 6 is regarded as low bias risk (32). At the same time, the Grading of Recommendations Assessment, Development and Evaluation (GRADE) system was used to evaluate the quality and strength of evidence for observational study (33). The included trials were divided into four grades, with the higher the grade, the higher the quality of the literature.

### 2.5 Statistical analysis

Statistical analyses were carried out using the software RevMan version 5.3 and Stata version 12.0. The multivariate-adjusted risk estimates (OR or HR values) from all the individual studies were collected to calculate the total effect sizes and 95% CIs *via* random effects model. A statistical heterogeneity analysis was performed by using Cochran’s Q statistic and the *I^2^* statistic (34). Significant heterogeneity was considered if the *P* value was<0.05 and we used the *I^2^* value to estimate the degree of heterogeneity, whereby 25%, 50%, and 75% represented low, medium and high heterogeneity, respectively (35).

The sources of heterogeneity were explored *via* sensitivity analysis, subgroup analyses and meta-regression analysis. A sensitivity analysis was performed to evaluate the effect of a specific study on the overall results by excluding one individual study at a time and combining the effect values of the remaining studies (36). Study designs (cohort studies and case control studies), region of subjects (Asia and Europe) and type of cancer (obesity related cancers and non-obesity related cancers) were considered when conducting subgroup analyses (37). Meta-regression analyses were carried out to quantitatively assess heterogeneity among the strata.

Potential publication bias was assessed using funnel plot symmetry and Egger’s test (38, 39). Trim and fill method was conducted to correct the result of bias and evaluate the impact of bias on the pooled risk estimates (40).

## 3 Results

4915 relevant articles were identified initially screened from electronic databases, but only 6 articles (992292 participants) met our inclusion criteria (**Figure 1**). These six articles included three cohort studies (27, 29, 41) with 862726 participants and three case-control studies (42–44) with 129566 participants. The detailed results are summarized in **Table 1**. Four of these studies were conducted in Asia (41–44), only two in Europe (27, 29). In addition, the outcome of four studies were obesity-related cancers (27, 41, 43, 44) and two were non-obesity-related cancers (29, 42). The risk of bias within included literature were evaluated *via* the NOS (**Tables 1**; **S3**). This average NOS score was 7.17 for all included studies, indicating high study quality. Simultaneously, the GRADE system was utilized to classify the quality of the included evidence. Its results were considered to be of medium quality (**Table 2**).

**Table 1:** Baseline characteristics of the participants.

Study	Country	Design	Characteristics of participants	Number of participants	Mean age (years)	Male (%)	TyG index analysis	Outcomes reported	Variables adjusted	NOS
Wang L, et al.	the United Kingdom	Cohort	General population aged 37-73 years	324334	55.8 ± 8.1	44.18	Categorized (median); Continuous	Lung cancer	Age, sex, region, Townsend deprivation score, smoking status, alcohol intake frequency, BMI, waist hip rate, hypertension, fasting time, TC, LDL-C, HDL-C and glycated hemoglobin	8
Okamura T, et al.	Japan	Cohort	General population	27921	45.7 ± 10.1	58.86	Continuous	Colorectal cancer	Sex, age, BMI, smoking status, alcohol consumption, exercise, SBP and serum creatinine	9
Fritz J, et al.	Norway, Sweden, Austria	Cohort	General population	510471	43.1 ± 10.6	50.54	Categorized (Q5:Q1); Continuous	Obesity-related cancer	Baseline age, sex, smoking status, fasting status, cohort and decade of birth and BMI	7
Yan X, et al.	China	Case-control	Outpatient	1578	—	42.97	Continuous	Non-small cell lung cancer	Age, sex, smoking, BMI, hypertension, WBCC, Neutrophil count, TC, LDL-C, HDL-C and uric acid	7
Kim YM, et al.	South Korea	Case-control	Outpatient	127564	48.6 ± 11.4	53.73	Categorized (Q4:Q1)	Gastric cancer	Age, sex and H. pylori infection	6
Panigoro SS, et al.	Indonesia	Case-control	Outpatient	424	—	—	Categorized (Q4:Q1)	Breast cancer	—	6

BMI, body mass index; TC, total cholesterol; LDL-C, low-density lipoprotein cholesterol; HDL-C, high-density lipoprotein cholesterol; SBP, systolic blood pressure; WBCC, white blood cell counts; TyG index, triglyceride glucose index; NOS, Newcastle-Ottawa scale; mean ± standard deviation.

**Table 2:** The Summary of Findings (SoF) with GRADE system.

Risk of cancer with different triglyceride glucose index levels.			
Population: Subjects with cancer vs. normal subjects/Subjects with high level of triglyceride glucose index vs. low level triglyceride glucose index.			
Settings: four studies were conducted in Asia, two study were conducted in Europe.			
Cases: Subjects with cancer/Subjects with high level of triglyceride glucose index.			
Controls: normal subjects/Subjects with low level of triglyceride glucose index.			
Outcomes	Effect size (95% CI)^a^	No of participants (studies)	Quality of the evidence Comments (GRADE)
Risk of cancer	1.14 (1.08,1.20)	992292 (six studies)	⊕⊕⊕ MEDIUM ^b^

GRADE working group grades of evidence.

High quality: We are very confident that the true effect lies close to that of the estimate of the effect.

Moderate quality: We are moderately confident in the effect estimate: The true effect is likely to be close to the estimate of the effect, but there is a possibility that it is substantially different.

Low quality: Our confidence in the effect estimate is limited: The true effect may be substantially different from the estimate of the effect.

Very low quality: We have very little confidence in the effect estimate: The true effect is likely to be substantially different from the estimate of effect.

Abbreviations: CI, confidence interval.

a Results for triglyceride glucose index levels of subjects with cancer compared with controls/Results for cancer risk of subjects with higher levels of triglyceride glucose index compared with lower triglyceride glucose index.

b Upgraded by one level because triglyceride glucose index was associated with cancer and all the results of the included studies were almost consistent (subjects with cancer had higher triglyceride glucose index).

**Figure 2:**
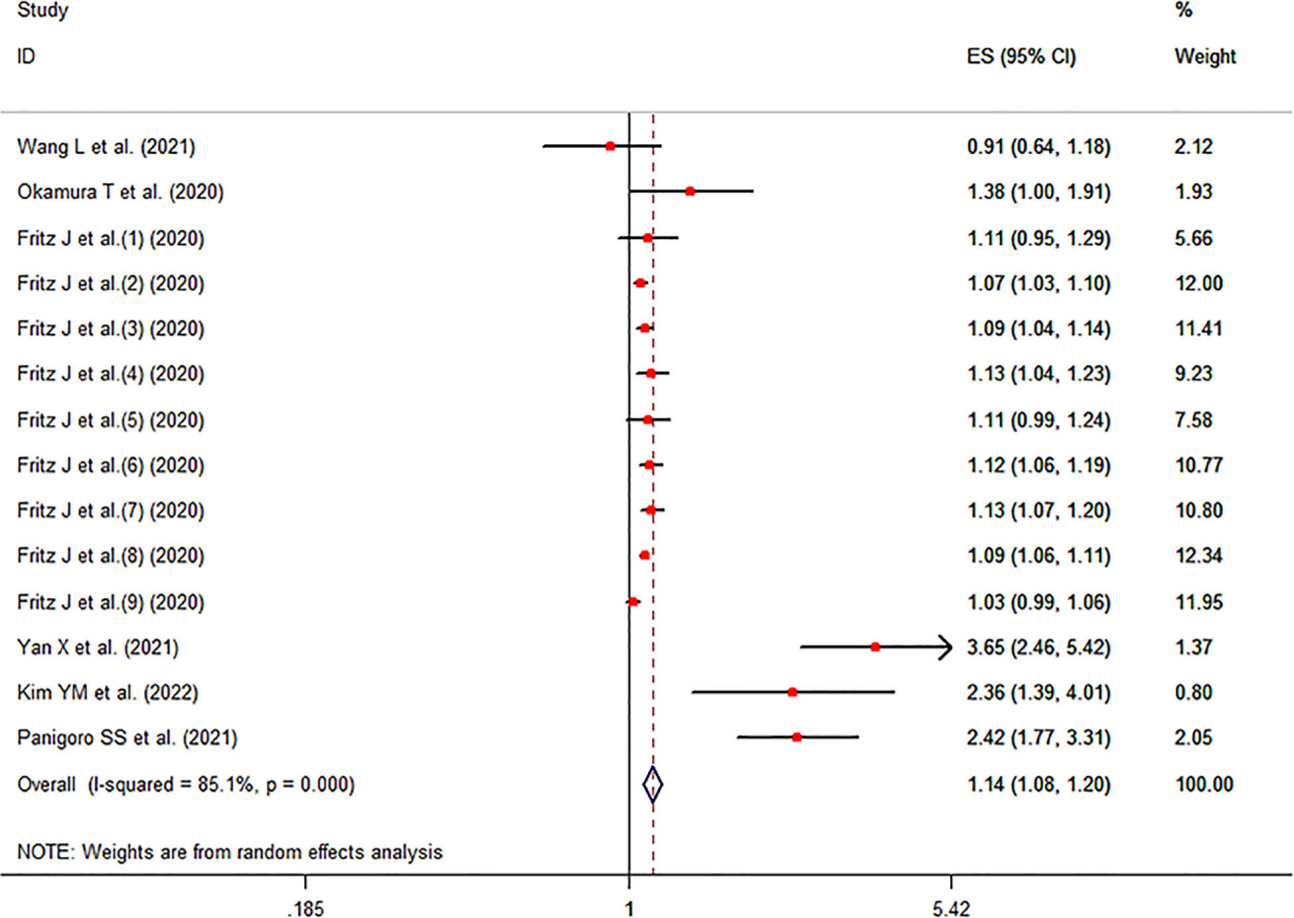
Forest plot of the cancer risk in subjects with high TyG index vs. control groups.

The meta-analysis showed that a higher TyG index increased the risk of cancer compared to a lower TyG index group (pooled effect size =1.14, 95% CI [1.08, 1.20], *I^2 =^* 85.1%, *P*<0.001, **Figure 2**).

**Figure 3:**
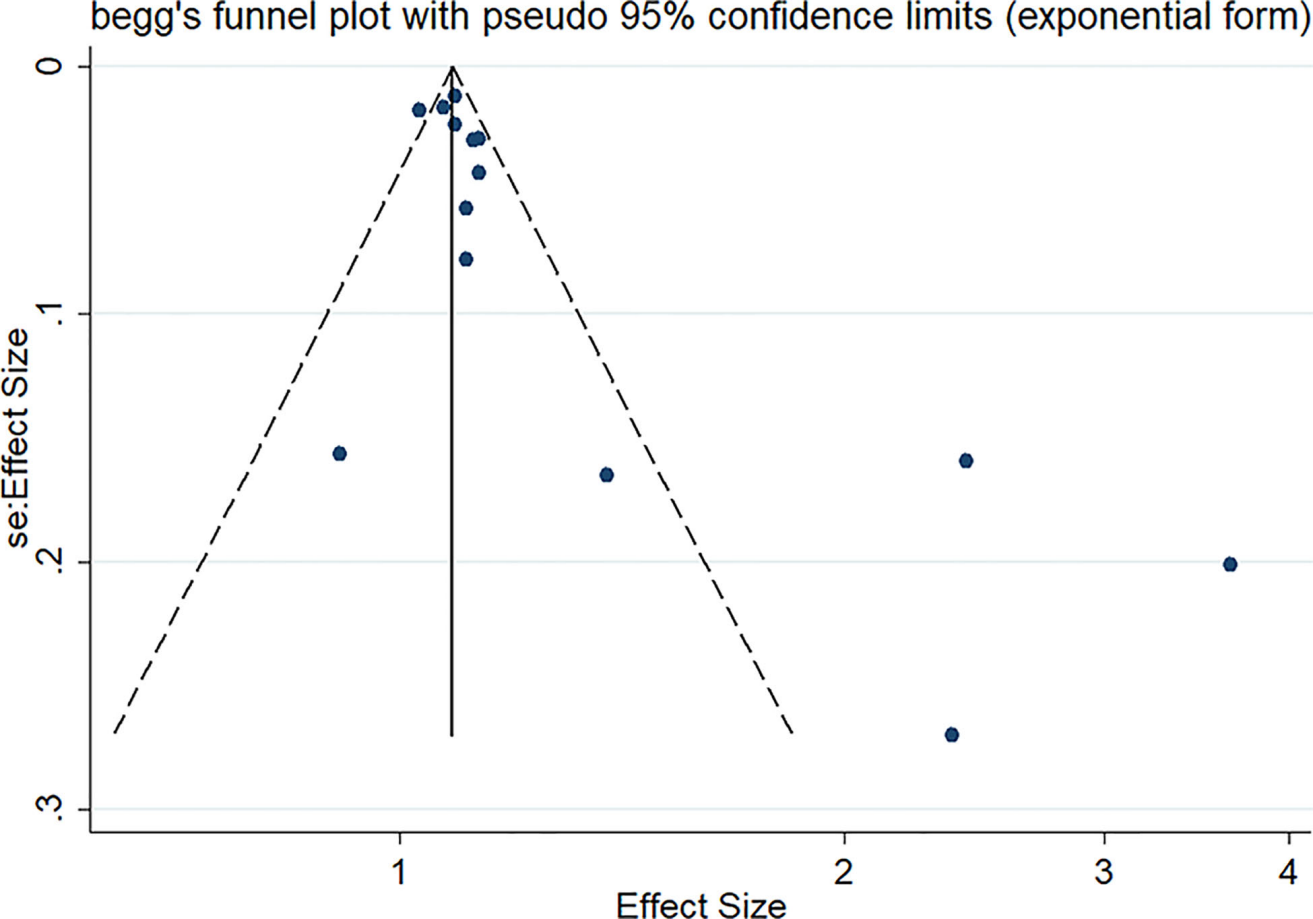
Funnel plot for the effect estimates of TyG index.

Sensitivity analysis indicated no extreme results affecting the pooled risk estimates (**Figure S1**). Meanwhile, the subgroup analyses were carried out in accordance with study design, region of subjects and cancer type. Cohort studies and case-control studies both showed a connection between TyG index and cancer (cohort studies: effect size =1.09, 95% CI [1.06,1.11], *I^2 =^* 41.9, *P*<0.001; case-control studies: effect size =2.76, 95% CI [2.09,3.65], *I^2 =^* 31.9, *P*<0.001). Moreover, in subgroup analyses of region, the TyG index was related to the risk of cancer in all results (Asia: effect size =2.29, 95% CI [1.51,3.49], *I^2 =^* 79.6, *P*<0.001; Europe: effect size =1.08, 95% CI [1.06,1.11], *I^2 =^* 40.1, *P*<0.001). Furthermore, the results of subgroup analysis in the light of the cancer type demonstrated that TyG index was associated with obesity-related cancers (effect size =1.11, 95% CI [1.07,1.16], *I^2 =^* 77.8, *P*<0.001). However, the relationship was not found in another subgroup. The specific details are shown in **Table 3**. The meta-regression identified study design and region as significant moderators for the cancer development (*P*<0.001). However, no similar effect between obesity-related cancers and non-obesity-related cancers was observed (**Table 4**). Therefore, we reasoned that heterogeneity might be caused primarily by study design and region factors based on the results of subgroup and meta-regression analyses.The funnel plot was asymmetric on visual inspection, indicating a high potential for publication bias (**Figure 3**). At the same time, the publication bias was also found by the Egger’s test (*P*=0.012). However, the effect size was no significant change after the trim and fill (adjusted: pooled estimate [95%CI]:1.083 [1.022,1.147], *P*=0.007, number of trim and fill=4), suggesting that the publication bias had little effect on the results.

**Table 3:** Results of subgroup analyses.

Subgrouped by	No.of studies	Effect size (95%CI)	*I^2^* (%)	*P*
Type of study	14	1.14(1.08,1.20)	85.1	<0.001
cohort Study	11	1.09(1.06,1.11)	41.9	<0.001
case-control study	3	2.76(2.09,3.65)	31.9	<0.001
Region	14	1.14(1.08,1.20)	85.1	<0.001
Asia	4	2.29 (1.51,3.49)	79.6	<0.001
Europe	10	1.08(1.06,1.11)	40.1	<0.001
Cancer type	14	1.14(1.08,1.20)	85.1	<0.001
Obesity-related cancers	12	1.11(1.07,1.16)	77.8	<0.001
Non-obesity-related cancers	2	1.81(0.47,7.07)	96.6	0.391

**Table 4:** Meta-regression for cancer incidence.

Variables	*I^2^* (%)	Adj R^2^	exp(b)	Std. Err.	*t*	*P*	95%CI
Study design (case-control studies)	40.41	99.51	2.53	0.30	7.91	<0.001	(1.96, 3.26)
Region (Asia)	59.68	99.47	2.03	0.26	5.52	<0.001	(1.54, 2.68)
Cancer type (non-obesity-related cancers)	84.87	5.46	1.42	0.43	1.16	0.27	(0.73, 2.76)

## 4 Discussion

With the rapid growth of cancer incidence and mortality, latest projections showed that cancer will become the main cause of death in countries around the world in the 21st century. Moreover, cancer has been confirmed to be closely related to IR in the current studies. However, as a valid predictor of IR, it is unclear whether cancer is related to TyG index. Our meta-analysis revealed that a higher TyG index was more likely to increase the likelihood of cancer compared to a lower TyG index group.

Although the specific mechanism of action of the TyG index on cancer has not been clarified, several potential mechanisms that could be related to IR or hyperinsulinemia have been proposed. First of all, IR syndrome has been considered as an important factor for cell proliferation (10, 19). Relevant studies have shown that hyperinsulinemia could affect energy metabolism by increasing the uptake of glucose by cells, and then activate certain signal transduction pathways in cells and directly increase the proliferation of cells and inhibit the apoptosis of cells, thereby promoting the occurrence of cancer (20, 45, 46). In addition, insulin likely plays a role in malignant transformation, cancer development and metastasis of various cells by binding to and activating its structurally related IGF-1 receptor (20, 21, 47). Moreover, high blood sugar itself also enhances the sensitivity of cells to IGF-1, which promotes the occurrence and development of cancer (48). Finally, IR triggers oxidative stress and inflammatory processes. Existing studies showed that IR indirectly promotes tumor growth through NF-κB and other pro-inflammatory signaling pathways (22). At the same time, abnormal blood sugar could increase oxidative stress, promote chronic inflammation, and then form a pro-angiogenic and anti-apoptotic microenvironment, which is an important cause of cancer (49, 50).

Our subgroup analyses demonstrated that high TyG index increased the risk of cancer in both Asia and Europe. However, the risk of developing cancer in Asia was about twice as high as in Europe. Ethnicity could be a crucial factor in this process (51, 52). Insulin secretion may itself be restricted in Asians relative to other regions (53). Moreover, Asian could consume more carbohydrate-containing foods than European, thereby increasing the likelihood of hypertriglyceridemia and impaired fasting glucose (54, 55) In addition, the phenomenon probably also was related to the level of social and economic development and rapid westernization of lifestyles (7, 56). Meanwhile, in our meta-analysis, considering the limited research in other regions, more relevant studies are needed in the future.

It has been widely accepted that obesity had a well-recognized association with cancer risk at various sites (57, 58). Moreover, our meta-analysis results also showed that TyG index was associated with cancer caused by obesity, and the cancer risk in high TyG index group was 1.11 times than that of lower level group. This could be attributed to the fact that obesity stimulates abnormal cell proliferation and inhibits cell apoptosis *via* adipocytokine activation (leptin and adiponectin), altered hormone metabolism, chronic (subclinical) inflammation, elevated insulin levels IGF-1 and other pathways, which negatively affects the incidence of cancer (27, 59–61). Although this relationship was not observed in the non-obesity related cancer group, some studies have demonstrated that higher fasting insulin levels and IR are associated with an increased risk of non-obesity-related cancers. The reasons may be that IR works through other pathways quite distinct from obesity (42, 62). Besides, limited by the number of studies, more studies are needed to explore its specific mechanism in the future.

Meanwhile, the high heterogeneity was observed in the results of our meta-analysis. Analyses of multiple methods demonstrated that research type and region could be attributed to the heterogeneity. This study also has several limitations. The studies included in the analysis were mainly observational studies, and its evidence level is lower than that of randomized controlled trials. In addition to the TyG index, residual confounding factors may also influence the relationship between cancer risk and TyG index, such as dietary and physical activity (63). Due to limitations in the number of studies, it’s unclear whether the TyG index increases cancer risk in a linear manner. Meanwhile, the sample sizes of contained studies varied widely, which may have unknown effects on the study results. Therefore, it is necessary to conduct more large-scale cohort studies and basic research, in order to obtain more conclusive evidence.

## 5 Conclusions

In summary, our meta-analysis indicated that higher TyG index may increase the risk of cancer. Considering the limitations of this meta-analysis, more prospective cohort studies and basic research are warranted to verify the relationship.

## Data availability statement

The original contributions presented in the study are included in the article/**Supplementary Material**. Further inquiries can be directed to the corresponding authors.

## Author contributions

HW and FY wrote and reviewed the manuscript; WC and GW made the meta-analysis design; HW, FY, YC, and FC carried out the specific study and analyzed the data. All authors contributed to the article and approved the submitted version.

## Supplementary material

Supplementary Figure 1 Sensitivity analysis for included studies.
