# Supplementary material for: Association between triglyceride glucose index and risk of cancer: A meta-analysis
Source: Front Endocrinol (Lausanne). 2023 Jan 12;13:1098492. doi: 10.3389/fendo.2022.1098492 (PMC9877418; doi:10.3389/fendo.2022.1098492)
Supplement: Supplementary file 2 [file Table_2.docx]

**Table S2** The specific search strategies

| #1 | tumor OR neoplasm OR tumors OR neoplasia OR neoplasias OR cancer OR cancers OR malignant neoplasm OR malignancy OR malignancies OR malignant neoplasms OR benign neoplasms OR benign neoplasm OR carcinoma OR carcinomas OR carcinomatosis OR carcinomatoses |
| --- | --- |
| #2 | triglyceride-glucose index OR triglyceride and glucose index OR TyG index OR triglyceride glucose index OR TyGs OR triglyceride glucose indices OR The triglyceride-glucose index OR Triglyceride/glucose index OR Triglycerides and glucose index |
| #3 | #1 AND #2 |
