# Supplementary material for: Association between triglyceride glucose index and risk of cancer: A meta-analysis
Source: Front Endocrinol (Lausanne). 2023 Jan 12;13:1098492. doi: 10.3389/fendo.2022.1098492 (PMC9877418; doi:10.3389/fendo.2022.1098492)
Supplement: Supplementary file 3 [file Table_3.docx]

**Table S3** The risk of bias by the Newcastle-Ottawa scale (NOS)

**Wang L, et al. 2021**

| Study type | cohort study | | |
| --- | --- | --- | --- |
| Participants | Participants aged 37 to 73 who entered the population-based national prospective cohort study.  Sample size: 324334  Mean age in years (SD): 55.831 (8.051)  Gender: 143277males/ 181057females  Location: the United Kingdom | | |
| Outcomes | Main study outcome: systematically examined the association between IR and lung cancer risk with IR measured by the TyG index and further examined the interactions and joint effects for lung cancer.  Available outcomes: triglyceride glucose index and lung cancer risk. | | |
| ***Risk of bias*** | | | |
| **Bias** | | **Authors’ judgment** | **Support for judgment** |
| Representativeness of the exposed cohort (**Selection**) | | 1 | truly representative of the average characteristics of the general population aged 37-70 in communities across the UK |
| Selection of the non exposed cohort (**Selection**) | | 1 | drawn from the same community as the exposed cohort |
| Ascertainment of exposure (**Selection**) | | 1 | secure record (eg laboratory examination) |
| Demonstration that outcome of interest was not present at start of study (**Selection**) | | 1 | yes  (no previous history of any type of cancer before enrollment) |
| Comparability of cohorts on the basis of the design or analysis (**Comparability**) | | 2 | study controls for age, gender, certain biochemical indicators and other factors |
| Assessment of outcome (**Outcome**) | | 1 | record linkage |
| Was follow up long enough for outcomes to occur (**Outcome**) | | 1 | yes (8.34-9.74 years) |
| Adequacy of follow up of cohorts (**Outcome**) | | 0 | no statement |

**Okamura T, et al. 2020**

| Study type | cohort study | | |
| --- | --- | --- | --- |
| Participants | Healthy participants aged 21 to 80 who entered the medical health checkup program.  Sample size: 27921  Mean age in years (SD): 45.7 (10.1)  Gender: 16434males/11487females  Location: Japan | | |
| Outcomes | Main study outcome: the association between triglyceride-glucose index and incident colorectal cancer  Available outcomes: triglyceride glucose index and incident colorectal cancer. | | |
| ***Risk of bias*** | | | |
| **Bias** | | **Authors’ judgment** | **Support for judgment** |
| Representativeness of the exposed cohort (**Selection**) | | 1 | truly representative of the average characteristics of the Japanese healthy population in community |
| Selection of the non exposed cohort (**Selection**) | | 1 | drawn from the same community as the exposed cohort |
| Ascertainment of exposure (**Selection**) | | 1 | secure record (eg health checkup program) |
| Demonstration that outcome of interest was not present at start of study (**Selection**) | | 1 | yes (apparently healthy Japanese men and women) |
| Comparability of cohorts on the basis of the design or analysis (**Comparability**) | | 2 | study controls for age, BMI and other factors |
| Assessment of outcome (**Outcome**) | | 1 | record linkage |
| Was follow up long enough for outcomes to occur (**Outcome**) | | 1 | yes (primary endpoint was set as incident colorectal cancer) |
| Adequacy of follow up of cohorts (**Outcome**) | | 1 | subjects lost to follow up unlikely to introduce bias (small number lost＜0.1%) |

**Fritz J, et al. 2020**

| Study type | cohort study | | |
| --- | --- | --- | --- |
| Participants | Participants from Metabolic Syndrome and Cancer Project (Me-Can), a pooling of six population-based cohorts.  Sample size: 510471  Mean age in years (SD): 43.1 (10.6)  Gender: 257968males/252503females  Location: Europe | | |
| Outcomes | Main study outcome: the role of insulin resistance as a mediator in the association of body mass index with site-specific cancer risk  Available outcomes: different level triglyceride glucose index and risk of cancer | | |
| ***Risk of bias*** | | | |
| **Bias** | | **Authors’ judgment** | **Support for judgment** |
| Representativeness of the exposed cohort **(Selection)** | | 1 | truly representative of the average in in the European community |
| Selection of the non exposed cohort **(Selection)** | | 1 | drawn from the same community as the exposed cohort |
| Ascertainment of exposure **(Selection)** | | 1 | secure record (eg laboratory examination) |
| Demonstration that outcome of interest was not present at start of study **(Selection)** | | 1 | yes (the earliest of first cancer diagnosis as endpoint) |
| Comparability of cohorts on the basis of the design or analysis **(Comparability)** | | 1 | study controls for age, gender, BMI and other factors. |
| Assessment of outcome **(Outcome)** | | 1 | secure record (data from national cancer registry, cause of death registry and population registry) |
| Was follow up long enough for outcomes to occur **(Outcome)** | | 1 | yes (median follow-up of 17.2 years) |
| Adequacy of follow up of cohorts **(Outcome)** | | 0 | no description of those lost |

**Yan X, et al. 2021**

| Study type | Cross-sectional study | | |
| --- | --- | --- | --- |
| Participants | Participants from the Department of Respiration of Nanjing Drum Tower Hospital.  Sample size:1578  Age: NA  Gender: 678males/900females  Location: China | | |
| Outcomes | Main study outcome: the association between triglyceride-glucose index and non-small cell lung cancer risk.  Available outcomes: different quantile triglyceride glucose index and incidence of non-small cell lung cancer. | | |
| ***Risk of bias*** | | | |
| **Bias** | | **Authors’ judgment** | **Support for judgment** |
| Is the case definition adequate(**Selection**) | | 1 | yes, with independent validation |
| Representativeness of the cases(**Selection**) | | 1 | consecutive or obviously representative series of cases |
| Selection of Controls(**Selection**) | | 0 | hospital controls |
| Definition of Controls(**Selection**) | | 1 | no history of disease (endpoint) |
| Comparability of cases and controls on the basis of the design or analysis(**Comparability**) | | 2 | study controls for age, gender and other factors |
| Ascertainment of exposure(**Exposure**) | | 1 | secure record (pathologically confirmed) |
| Same method of ascertainment for cases and controls(**Exposure**) | | 1 | yes |
| Non-Response rate(**Exposure**) | | 0 | non respondents described |

**Kim YM, et al. 2022**

| Study type | Cross-sectional study | | |
| --- | --- | --- | --- |
| Participants | Patients undergoing upper gastrointestinal endoscopy at the medical center.  Sample size: 127564  Mean age in years (SD): 48.6 (11.4)  Gender: 68536males/59028females  Location: Korea | | |
| Outcomes | Main study outcome: the association between the triglyceride-glucose index and gastric carcinogenesis, including precancerous conditions  Available outcomes: the relationship between increase of TyG index and gastric cancer | | |
| ***Risk of bias*** | | | |
| **Bias** | | **Authors’ judgment** | **Support for judgment** |
| Is the case definition adequate(**Selection**) | | 1 | yes, with independent validation |
| Representativeness of the cases(**Selection**) | | 1 | consecutive or obviously representative series of cases |
| Selection of Controls(**Selection**) | | 0 | hospital controls |
| Definition of Controls(**Selection**) | | 1 | no history of disease (endpoint) |
| Comparability of cases and controls on the basis of the design or analysis(**Comparability**) | | 1 | study controls for any additional factors |
| Ascertainment of exposure(**Exposure**) | | 1 | secure record (laboratory examination) |
| Same method of ascertainment for cases and controls(**Exposure**) | | 1 | yes |
| Non-Response rate(**Exposure**) | | 0 | non respondents described |

**Panigoro SS, et al. 2021**

| Study type | Cross-sectional study | | |
| --- | --- | --- | --- |
| Participants | Individuals aged 19 years or above in six public referral hospitals in Indonesia.  Sample size:424  Age: NA  Gender: 424 females  Location: Indonesia | | |
| Outcomes | Main study outcome: the association and dose-response between triglyceride-glucose index and breast cancer.  Available outcomes: different quantile triglyceride glucose index and breast cancer. | | |
| ***Risk of bias*** | | | |
| **Bias** | | **Authors’ judgment** | **Support for judgment** |
| Is the case definition adequate(**Selection**) | | 1 | yes, with independent validation |
| Representativeness of the cases(**Selection**) | | 1 | consecutive or obviously representative series of cases |
| Selection of Controls(**Selection**) | | 0 | hospital controls |
| Definition of Controls(**Selection**) | | 1 | no history of disease |
| Comparability of cases and controls on the basis of the design or analysis(**Comparability**) | | 1 | study controls for age and other factors |
| Ascertainment of exposure(**Exposure**) | | 1 | secure record (Laboratory examination) |
| Same method of ascertainment for cases and controls(**Exposure**) | | 1 | yes |
| Non-Response rate(**Exposure**) | | 0 | non respondents described |
