# Supplementary figures and images for: Association between triglyceride glucose index and risk of cancer: A meta-analysis
Source: Front Endocrinol (Lausanne). 2023 Jan 12;13:1098492. doi: 10.3389/fendo.2022.1098492 (PMC9877418; doi:10.3389/fendo.2022.1098492)

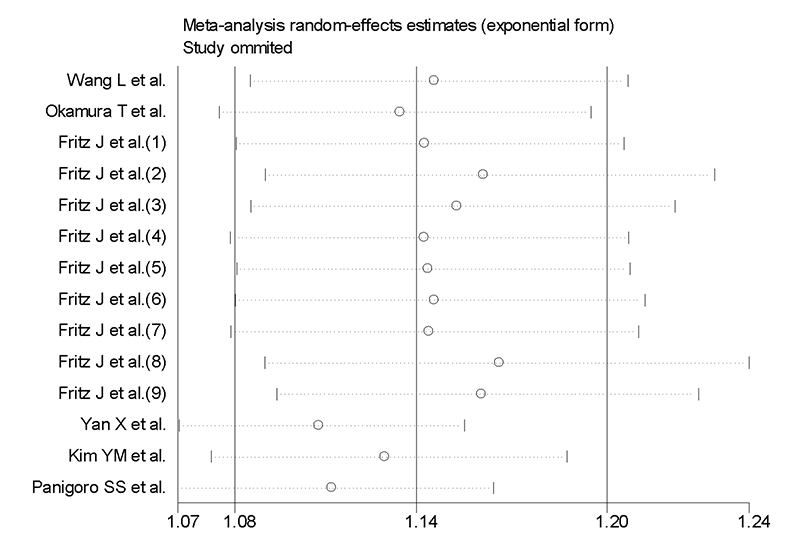

Supplement: Supplementary Figure 1 — Sensitivity analysis for included studies. [file Image_1.tif]
